# Bioactivated scaffolds promote angiogenesis and lymphangiogenesis for dermal regeneration in vivo

**DOI:** 10.1177/20417314251317542

**Published:** 2025-03-12

**Authors:** Benedikt Fuchs, Sinan Mert, Daniel Hofmann, Constanze Kuhlmann, Alexandra Birt, Paul Severin Wiggenhauser, Riccardo E Giunta, Myra N Chavez, Jörg Nickelsen, Thilo Ludwig Schenck, Nicholas Moellhoff

**Affiliations:** 1 Division of Hand, Plastic and Aesthetic Surgery, LMU University Hospital, LMU Munich, Munich, Germany; 2 Molecular Plant Science, Department Biology I, LMU Munich, Munich, Germany; 3 Institute of Anatomy, University of Bern, Bern, Switzerland; 4 Praxis Dr. Schenck, Munich, Germany

**Keywords:** Cyanobacteria, hyaluronic acid, oxygen, biomaterials

## Abstract

Chronic wounds represent an unresolved medical challenge with significant impact for patients’ quality of life and global healthcare. Diverse in origin, ischemic-hypoxic and inflammatory conditions play central roles as pathological features that impede proper tissue regeneration. In this study, we propose an innovative approach to address this challenge. Our novel strategy utilizes photosynthetic biomaterials to restore the wound healing process firstly by promoting a normoxic, regeneration-supporting environment and secondly by mitigating inflammation through restoring lymphatic fluid transport and improving blood perfusion. We designed bioartificial scaffolds with photosynthetic cyanobacteria (Syn*echococcus sp. PCC* 7002) and assessed their functional integration in a bilateral full-thickness skin defect on the backs of mice over a period of 7 days. Illuminated photosynthetic cyanobacteria facilitated local tissue oxygenation independent of blood perfusion. Additionally, genetic modification enabled the secretion of lymphangiogenic hyaluronic acid (HA) into the wound area. After 7 days, the scaffolds were explanted and histologically examined, assessing cell migration (HE staining) and protein expression (CD31, LYVE-1, VEGFR3, Ly6G, and F4/80). Results demonstrated successful colonization of bioartificial scaffolds with cyanobacteria. Following implantation into bilateral full-thickness skin defects, we observed an adherent vascularized basal layer beneath the bioactivated scaffolds after 7 days. Substantial increases in cell migration within bacteria-loaden scaffolds were noted, accompanied by a heightened expression of lymphatic (LYVE-1 and VEGFR3) and endothelial cell markers (CD31). Simultaneously, an augmented expression of acute (Ly6G) and late (F4/80) inflammatory proteins was observed. In summary, we developed a viable photosynthetic scaffold by integrating cyanobacteria into dermal regeneration materials (DRM), promoting the expression of lymphatic, endothelial, and inflammatory proteins under hypoxic conditions. The findings from this study represent a significant advancement in establishing autotrophic tissue engineering approaches, advocating for the use of photosynthetic cells in treating a broad spectrum of hypoxic conditions.

## Graphical abstract

**Graphical Abstract:**
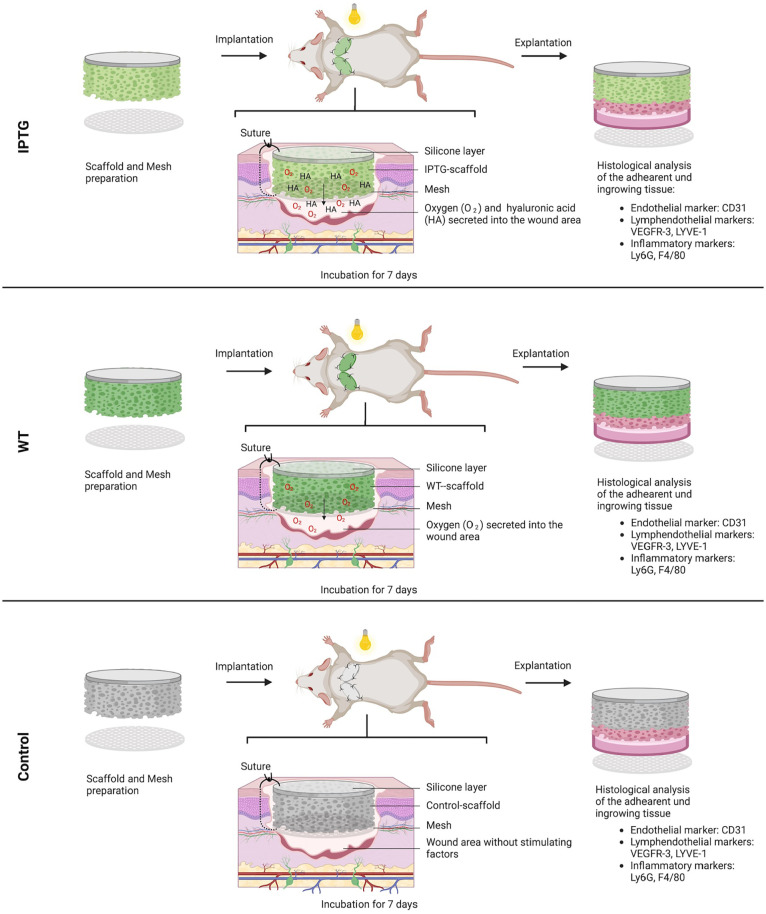

## Introduction

Chronic wounds remain a persistent challenge within the medical field today. Approximately 1%–2% of the population will experience a chronic wound during their lifetime.^1^ Within industrialized nations, an average of 2.21 per 1000 individuals endure chronic wounds.^2^ Notably, in the United States, where an estimated 6.5 million people struggle with chronic skin ulcers, a phenomenon termed a “silent epidemic” has been observed.^3,4^ These wounds exert significant socioeconomic ramifications on both the state and society at large, with the collective annual treatment costs of chronic skin defects reaching 25 billion US dollars in the USA.^3^

Pathophysiologically, chronic wounds denote a disruption in one or more phases of the wound healing cascade. The wound healing process is predominantly entrenched within the inflammatory phase, whereby the initially self-limiting inflammation transitions into a self-perpetuating state. Notably, tissue hypoxia assumes a pivotal role in this process. In chronic wounds, a diminished oxygen content ranging from 5 to 20 mmHg (pO_2_) has been shown due to compromised perfusion conditions, in contrast to control tissue exhibiting levels of 30–50 mmHg (pO_2_).^5^ Oxygen serves as a critical mediator in various wound healing processes, crucially facilitating re-epithelialization, fibroblast proliferation, and collagen synthesis.^6,7^ Studies indicate that active fibroblast cell division is only observable at pO_2_ levels exceeding 15 mmHg.^8^ Suboptimal oxygen partial pressures hinder the binding affinity of growth factors to dermal fibroblasts, consequently diminishing collagen synthesis.^9,10^ In addition to oxygen, the effective removal of phagocytes and pro-inflammatory debris from the wound site via the lymphatic system also assumes a pivotal role in regulating inflammation and facilitating tissue regeneration.^11,12^ The failure to expeditiously eliminate resultant degradation products via the lymphatic system exacerbates inflammation.^13^ This hostile microenvironment precludes the transition to a proliferative healing phase and impedes tissue regeneration. Overall, chronic wounds’ regenerative potential is curtailed by an inadequacy of growth factors and oxygen within the wound bed. This cytotoxic milieu fosters insufficient lymphangiogenesis, vascularization, and wound healing.

Presently, autologous skin grafts represent the gold standard for treating full-thickness skin defects.^14^ Nonetheless, this approach is marred by restricted availability and donor site morbidity. Consequently, researchers are keenly interested in devising novel strategies to deliver acids and growth factors to the wound site while concurrently fostering inflammation. Given this imperative, there exists a pressing need for more sophisticated wound dressings tailored to the intricacies of wound healing physiology and chronicity.^14^ Hence, tissue engineering is assuming rising significance with the advent of biological 3D scaffolds designed to mechanically support vascular invasion, cell migration, and proliferation in tissue regeneration.^15,16^

In the realm of tissue engineering, there has been a discernible inclination toward the utilization of naturally derived substances. Notably, microorganisms, including microalgae and cyanobacteria, have attracted significant global attention for their potential in chronic wound healing. Leveraging their photosynthetic capabilities, these microorganisms present a viable source of localized oxygen, circumventing reliance on blood perfusion to alleviate tissue hypoxia.^17–20^ Furthermore, they can be subjected to genetic modification to facilitate the secretion of recombinant growth factors directly into the wound site.^21^ Given the light-dependent nature of these bacteria, their strategic deployment proves particularly advantageous for dermal applications on bodily surfaces.

Thus, we have devised a collagen-based three-dimensional scaffold seeded with photosynthetic cyanobacteria of the strain *Synechococcus* sp. PCC 7002 to create a bioactivated matrix. Zhang et al.^22^ conducted genetic modification through the transfection of hyaluronic acid synthetases into these bacteria, thereby establishing them as a foundational element in a biotechnological platform for the production of recombinant factors. The incorporation of an IPTG (Isopropyl beta-D-thiogalactoside) promoter facilitates the synthesis and extracellular release of bacterial hyaluronic acid, a vital constituent of the extracellular matrix.^23^ Hyaluronic acid exerts a significant influence on tissue and wound healing processes by promoting lymphangiogenesis^23^ and angiogenesis,^24,25^ activating inflammatory cells,^26–28^ and facilitating fibroblast migration.^29,30^ In our preliminary investigations, we observed notable effects of the bioactivated scaffolds on the expression of lymphatic markers at both gene and protein levels, as well as on proliferation characteristics and lymph vessel formation.^31^ Moreover, our experiments demonstrated sustained oxygen production over a span of 7 days. Notably, cytotoxicity assessments conducted in vitro on human dermal cells revealed no adverse effects attributable to the bacteria. Nonetheless, further in vivo investigations are imperative to elucidate their overall impact on wound regeneration, thereby validating their feasibility and efficacy.

## Materials and methods

### Cell culture of cyanobacteria

For the experiments two types of cyanobacteria were used: Wild-type (SynWT) and transgenic Synechococcus sp. PCC 7002 cyanobacteria (strain SynHA12) a kind gift from Jörg Nickelsen, Molecular Plant Science, Department Biology I, LMU Munich, Munich, Germany.^22^ The genotype of the SynHA12 strain is: Δ*glgA1*: : Pcpt*-glmS-glmU-CmR*+Δ*AcsA*: : PcLac143*-pmHAS*. Both types were culti-vated on A-D7 medium agar-plates supplemented with glucose (1 g/L) and chloramphenicol (10 μg/mL) at 30–50 μE m^−1^ s^−1^ and 25°C–30°C. Plates were refreshed every 3 weeks. A liquid preculture was started before each experiment by inoculating agar-growing cyanobacteria in 50 mL A-D7 medium (supplemented with 1 g/L glucose) and incubating it for 3 days at standard culture conditions (30°C, 150 rpm, 30–50 μE m^−1^ s^−1^). The transgenic strain overexpresses the *Pasteurella multocida* hyaluronic acid synthetase, which enables hyaluronic acid production and secretion.^22^ For inducing hyaluronic acid production, 1 mM IPTG (Isopropyl beta-D-thiogalactoside, Merck, Darmstadt, Germany) was added to the mutated cyanobacteria cultures resuspended in fresh medium to OD750 = 1 (IMPLEN P300 Nanophotometer, Munich, Germany) and further cultured for at least 7 days (IPTG) The wild type bacteria (WT) were cultured under the same conditions as a strain without gentic modification. Detailed culture conditions have been described previously.^31^ For all cell-culture experiments, cyanobacteria cell number was determined by light-microscopy (Primovert, Zeiss, Oberkochen, Germany) using a Neubauer cell-chamber. Cyanobacteria were then cultured under standard mammalian cell culture conditions (37°C, 5% CO2) and constant illumination by placing them under a light source with the complete spectrum of white light at a distance of 25 cm above the samples (32.25 μE m^−1^ s^−1^, LED, Sebson, Dortmund, Germany) to allow photosynthetic growth.

### Seeding of cyanobacteria in DRM

Integra bilayer matrix wound dressing (IDRT, Integra©Matrix Life Science Cooperation, Plainsboro, NJ, USA) composed of collagen and silicon layers was used as a dermal scaffold. Bacterial seeding was performed as described previously.^31,32^ In brief, for all experiments, scaffolds were cut using a Ø12 mm biopsy punch (Pico Punch^®^P1225, Acuderm^®^Inc., Ft. Lauderdale, FL, USA), air-dried for 20 min on sterile gauze, and placed with the collagen-layer facing upwards in 6-well plates. We used a double-layered scaffold where the collagen is covered with a silicone layer on top, which acts as a temporary epidermis. Unless stated otherwise, cyanobacteria were seeded at a final density of 1 × 10^7^ cells per scaffold. For bacteria seeding into the scaffolds, bacteria were transferred into a 50 mL tube and centrifuged at 4000*g* for 5 min. Thereafter, the supernatant was discarded, the bacteria were washed with 50 mL PBS, and the suspension was centrifuged at 4000*g* for 5 min. Subsequently, the pellet was resuspended in 50 μL medium. Next, 50 μL Fibrin (TISSEEL, Baxter GmbH, Unterschleißheim, Germany) in a ratio of 1: 1 were added, and the solution was pipetted onto the scaffold. Lastly, to ensure fixation of seeded bacteria within the scaffold 50 μL thrombin solution (TISSEEL, Baxter GmbH, Unterschleißheim, Germany) was pipetted into the scaffold to seal the bacteria within it. Scaffolds were air-dried for 1 h and then cultured with A-D7 medium, covering the scaffold at 30°C and 5% CO_2_ under constant illumination for the desired time. We prepared scaffolds with genetically mutated bacteria with the addition of promoter IPTG (IPTG) and non-mutated wild-type bacteria (WT), as described previously. As a negative in vivo control group (KO), we prepared empty scaffolds containing A-D7 medium without integration of cyanobacteria.

### Stereoscopy and microscopy

Before the scaffolds were implanted, images were taken after 7 days of bacterial colonization using stereoscopes (Primovert, Zeiss, Oberkochen, Germany). Immediately after scaffold implantation, the scaffolds were analyzed microscopically (Primovert, Zeiss, Oberkochen, Germany). Transillumination enabled the detection of vascularization within and directly adherent to the scaffolds. The images obtained were then further analyzed using the VegSeg tool. The proportion of the vascularized area to the total scaffold area was determined and compared.

### Full-skin defect model

For in vivo experiments, scaffolds were prepared as described previously. Before implantation, we prepared three scaffold groups: IPTG, WT, and control scaffolds (KO). Scaffolds were incubated in A-D7 -medium for 3 days under previously described conditions. The experimental procedure of the present study was approved by the District Government of Upper Bavaria (ROB-55.2-2 532.Vet_02–19-96) and performed according to the current German Animal Welfare Act (TierSchG). Experiments were performed on female nude mice aged 6–8 weeks and with body weights of 20–25 g (Charles River, Sulzfeld, Germany). Under inhalation anesthesia (Isoflurane, Baxter Germany, Unterschleissheim, Germany), a bilateral 10 mm full-skin defect was created. Scaffold implantation in mice was previously published in detail.^19^ Control, IPTG, and WT scaffolds were implanted and covered with a transparent dressing (V.A.C. Drape, KCI Medical Products, Wimborne Dorset, UK). In order to trigger the oxygen production, cages were equipped with a flexible LED module by placing them under a light source with the complete spectrum of white around the cages (32.25 μE m^−1^ s^−1^, LED, Sebson, Dortmund, Germany) to stimulate the photosynthetic scaffolds during daytime. Mice were kept in the Central Animal Facility at the Medical Faculty of the Ludwig-Maximilian-University Munich under 12 h light/dark cycles, with constant temperature, in individually ventilated cages. Thus, photosynthetic implants could not be exposed for more than 12 h to light stimulation per day. Control animals were kept under the same light stimulation conditions. At 7 days after implantation, mice were euthanized by cervical vertebrae dislocation. The skin from the back, including the scaffolds, was excised for further analysis. Subsequently, scaffolds were fast-frozen in liquid nitrogen and stored at −80°C.

### Paraffin embedding

First, sections were placed in collection cassettes (Histosette^®^2 with Lid; Simport; Bernand-Pilon; Canada) and stored in 100% isopropanol (Otto Fischar GmbH & Co. KG; Saarbrücken). The collection cassettes were loaded into a vacuum infiltration tissue processor (Tissue-Tek^®^VIP^®^6; Sakura Seiki Co. Ltd, Nagano, Japan). The following ascending alcohol series was used for dehydration at 40°C: Neutral buffered formaldehyde 10% (Otto Fischar GmbH & Co. KG; Saarbrücken) for 1 h, Ethanol 70% for 30 min, Ethanol 70% for 1 h, Ethanol 96% for 1 h (repeated twice), Ethanol 100% for 1 h (repeated thrice). The fixation was done using Tissue Clear^®^ (xylene substitute; Sakura Seiki Co. Ltd, Nagano, Japan; REF: 1426) for 2 × 1 h at 40°C. The embedding was performed with Tissue Tek^®^ Paraffin Wax (TEK 3 Polymer; Sakura Seiki Co. Ltd, Nagano, Japan; REF 451) at 63°C for 30 min, followed by 1 h, and then 2 × 30 min. Sections were embedded in paraffin using a casting machine (AGS-W; RWW Medizintechnik) with Tissue Tek^®^ Paraffin Wax (TEK 3 Polymer; Sakura Seiki Co. Ltd, Nagano, Japan; REF 451) in prefabricated molds. Paraffin blocks were solidified on a cooling plate at −15°C (Para-Cooler-T.W.; RWW Medizintechnik) and sectioned into 5 µm slices using a rotary microtome (Microm HM 355S; Thermo Scientific) with blades (Feather, Microtome Blades N35 Type, Japan). Next, sections were floated in a tissue flotation bath (TFB 45; Medite Medizintechnik) and mounted onto slides. Wet slides were placed on a stretching plate at 68°C (Meda X) to remove folds. The slides were baked in an oven at 70°C (Memmert GmbH & Co. KG, Schwabach) to complete the process.

### Chromogenic immunohistochemical staining

This study involves detailed immunohistochemistry (IHC) staining of mouse skin sections and skin substitute materials (scaffolds) using various polyclonal antibodies to visualize specific antigens. The antibodies used include LYVE-1, VEGFR3, CD31, Ly6g, F4/80. An anti-rabbit secondary antibody conjugated with HRP and DAB was employed for detection. First, sections were immersed twice for 10 min each in xylol to remove paraffin. Then, sections were rehydrated through a graded ethanol series (100%, 95%, 80%, and 60%), each for 2 min, and rinsed three times for 3 min each in tap water and once in TBS buffer. Then, sections were subjected to heat-induced epitope retrieval using a citrate buffer (0.01 M, 4°C) at pH 6.0, boiled at 95°C, and then cooled for 10 min. For blocking endogenous peroxidase activity, sections were incubated in 3% H₂O₂ in methanol for 30 min at room temperature to block endogenous peroxidase activity. A blocking solution of 5% BSA in TBS-T (0.1% Tween 20) was prepared, and sections were incubated in this solution for 20 min to prevent non-specific binding of antibodies. Primary antibodies (Table 1) were diluted in the blocking solution according to the manufacturer’s instructions. Then, 200 μL of the diluted primary antibody solution was applied to each section, which was then incubated overnight at 4°C. Sections were washed three times for 3 min each in TBS and incubated with a biotinylated anti-IgG (H+L) for 10 min at room temperature, followed by washing. For the secondary antibody application, we used the IHC polymer detection kit HRP/DAB (ab64261) according to the manufacturer’s instructions. Streptavidin peroxidase was applied and sections were incubated for 10 min at room temperature, followed by four washes in TBS buffer. A DAB solution was prepared by mixing 20 μL DAB chromogen with 1 mL DAB substrate and applied to sections for 10 min at room temperature for color development. Next, sections were counterstained with hematoxylin for 1 min to visualize cell nuclei blued in tap water and mounted with coverslips for microscopic examination. The stained images were then analyzed with Image-J, and the number of cells per mm^2^ or vessels per area in μm^2^ was determined.

**Table 1.** Primary antibodies.

Name	References	Isotype and host species	Dilution
LYVE-1	ab14917	IgG rabbit	1: 100
VEGFR3	ab27278	IgG rabbit	1: 100
CD31	ab28364	IgG rabbit	1: 40
Ly6g	ab238132	IgG rabbit	1: 100
F 4/80	(ab111101)	IgG rabbit	1: 500

### Statistical analysis

All assays were performed in at least three independent experiments with at least two technical replicates in each experimental group. All data are presented as mean ± standard deviation. Student’s *t*-test was performed for comparison between two different groups. Differences among groups were considered significant if *p* ⩽ 0.05 (ns: not significant; *p* ⩽ 0.05; **p ⩽ 0.01; ***p ⩽ 0.001; ****p ⩽ 0.0001). All data were tested for normal distribution before performing a t-test. Schematic representations were created using the platform www.BioRender.com.

## Results

### Adherent vascularized basal layer of the scaffolds

**Figure 1.**
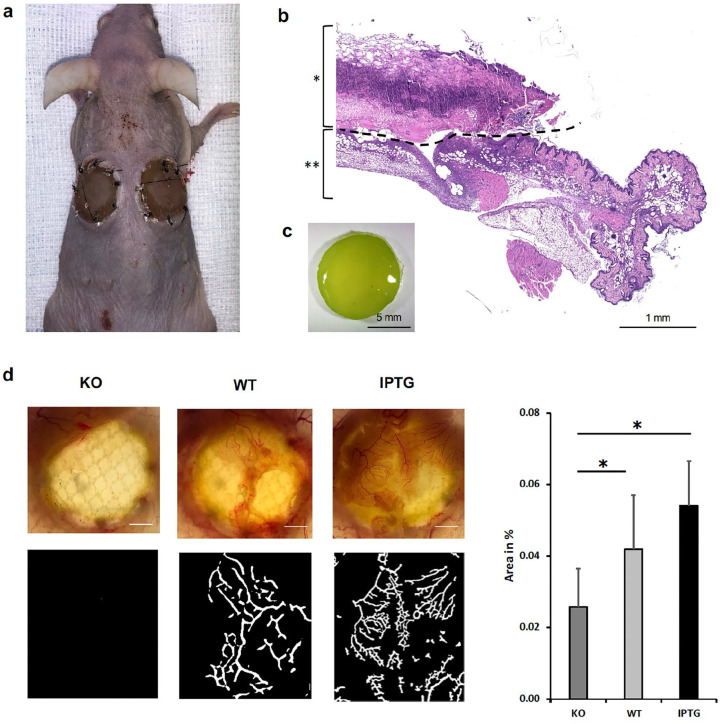
(a) Representative image of implanted scaffolds in a bilateral full-skin defect on mice after 7 days before the skin is harvested. (b) The cross-sectional image of an explanted scaffold in H&E staining after 7 days. The picture was taken using a brightfield microscope (Axio Observer, Zeiss, Oberkochen, Germany), Magnification 10×. Scale bar represents 1 mm. The black dashed line marked the scaffolds boundary and transition into the surrounding tissue. The scaffold is marked with (*). (**) shows the adjacent and ingrowing surrounding tissue. (c) Representative picture of a seeded scaffold bevor implantation. The overall green color of the scaffold demonstrates a homogeneous distribution of cyanobacteria throughout the scaffold-area. After 7 days, scaffolds were implanted as described previously. Scale bar represents 5 mm. (d) Transillumination directly after scaffold explantation showed an ingrowing vascularized adherent basal layer in bioactivated scaffolds compared to control scaffolds with absence of bacteria. We quantified the vessel formation with VesSeg tool as area in %. Scale bar represents 2 mm. (*N* = 12, *p ⩽ 0.05).

To observe the effect of bioactivated scaffolds on wound regeneration, Syn-HA scaffolds were implanted in a bilateral full-thickness skin defect (Figure 1(a)) on the back of mice for 7 days. The localization at the back was chosen for sufficient illumination of the scaffolds and for not being removable by the mice themselves. Figure 1(c) shows a representative image bevor implantation. After 7 days, the scaffolds were explanted and transilluminated to detect sprouting vascularized tissue. Figure 1(b) shows a cross-sectional image after the explantation. We observed a basal adherent vascularized layer, particularly in the bioactivated scaffolds, which appeared to have grown into the scaffold (Figure 1(d)). This tissue layer was almost absent in the corresponding control without the presence of bacteria. Using the Veg-Seg tool, we quantified the area of vessels in percent, although one should note that morphologically only blood vessels can be detected at this level and this does not preclude the existence of a lymphatic network (Figure 1(d)). We detected a significant vascular network in bioactivated scaffolds compared to the control scaffolds. The scaffolds with stimulated transgenic bacteria did not show a markedly increased area of vessels compared to the wild-type bacteria. This suggests that the vascularized tissue mainly benefited from the bacterial photosynthetically produced oxygen. The scaffolds obtained were processed by H&E staining for further histological examination.

### Cell migration into scaffolds

**Figure 2.**
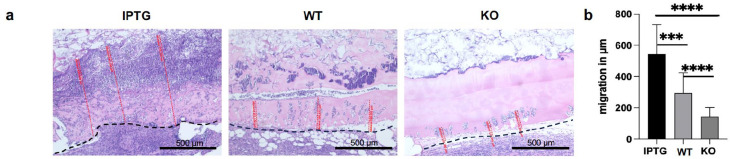
(a) Histological H&E staining of cell migration into scaffolds colonized with either (a) IPTG simulated transgenic cyanobacteria (IPTG) or wild-type bacteria (WT). The control scaffolds contained no bacteria (KO). The scale bar represents 500 μm. Representative pictures are shown. The black dashed line marked the scaffolds boundary and transition into the surrounding tissue. (b) Quantification of the migration depth in µm into the scaffolds (*N* ⩾ 10; ***p ⩽ 0.001; ****p ⩽ 0.0001).

Next, we investigated the impact of the secretion of the growth factor hyaluronic acid and the oxygenated environment on the migration of cells into the scaffold. The scaffolds should provide a significantly more growth-friendly and attractive environment for cell growth. For this purpose, the bioactivated scaffolds were implanted on the back of mice for 7 days as described above and the penetration depth of dermal cells into the scaffold was measured after histological preparation (Figure 2(a) and (b)). A significantly higher average migration depth of 557.34 ± 214.2 µm was measured in the scaffolds stimulated with IPTG compared to 295.6 ± 133.5 µm in the scaffolds colonized with wild-type bacteria (WT). The control scaffold (KO) showed only slight cell migration with the absence of bacteria and a cell penetration depth of 171.1 ± 55.4 µm. These results indicate a significant influence of secreted hyaluronic acid as a cell-attractant for positive chemotaxis in vivo.

### Detection of cell migration of lymphatic cells using LYVE1 and VEGFR-3

**Figure 3.**
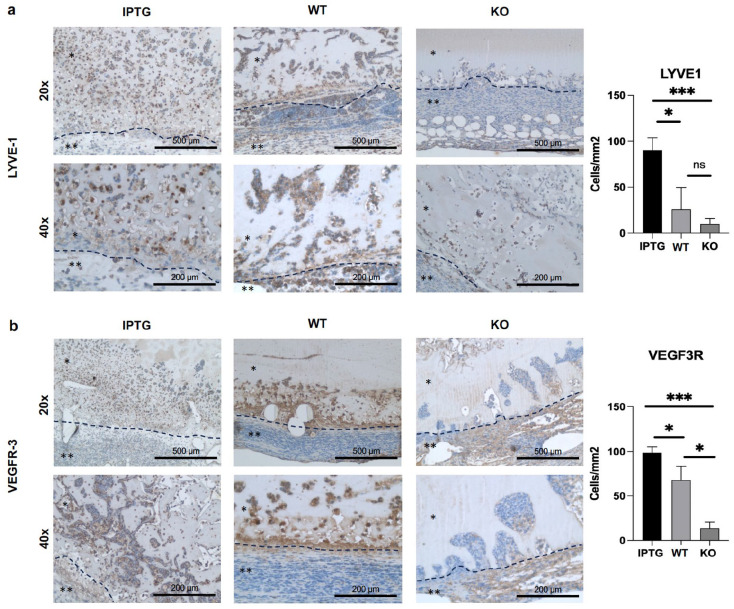
(a) Immunohistochemical staining of endothelial vessel density in scaffolds colonized with either IPTG-induced transgenic cyanobacteria (IPTG) or wild-type bacteria (WT). Control scaffolds (KO) contained no bacteria. The scale bar represents 500 μm at 20× magnification and 200 μm at 40× magnification. Representative images are provided. The mark with one star indicates the scaffold. Two stars mark the adherent ingrowing tissue layer. The detected vessels are marked with an arrow as an example. Vessel area quantification was performed relative to the total area per square millimeter in CD31-stained scaffolds, analyzed using ImageJ software. The black dashed line marked the scaffolds boundary and transition into the surrounding tissue. Statistical analysis was conducted (*N* = 10, ns *p* > 0.05; *p ⩽ 0.05; **p ⩽ 0.01).

Subsequently, we evaluated the regenerative potential of the bioactivated scaffolds within the wound model. Following application as previously outlined, the scaffolds were extracted and subjected to histological analysis. The primary focus centered on assessing the scaffolds’ impact on lymphangiogenesis stimulation. To accomplish this objective, sections were immunohistochemically stained for typical lymphatic markers LYVE-1 and VEGFR-3 to identify lymphatic cells (Figure 3(a) and (b)). The marked cells were detected at 20× and 40× magnification. The quantification of lymphoid cell infiltration into the scaffold was based on the staining patterns observed at 20× magnification and analyzed by Image-J as cells per mm^2^. A notably elevated cell density per square millimeter expressing the LYVE-1 receptor was observed in transgenic scaffolds (IPTG; 92 ± 28 cells/mm^2^), compared to scaffolds colonized with wild-type bacteria (23 ± 17 cells/mm^2^). However, no significant difference was observed between scaffolds colonized with wild-type bacteria and the control, indicating that the secreted hyaluronic acid plays a pivotal role in chemotaxis. This finding was corroborated by VEGFR-3 staining, revealing a significantly heightened cell density per square millimeter of lymphoid cells within the IPTG scaffolds (98 ± 6 cells/mm^2^) compared to those with wild-type bacteria (64 ± 14 cells/mm^2^) and the control (14 ± 7 cells/mm^2^). In summary, these findings elucidate a distinct stimulatory effect of secreted hyaluronic acid on the migration of lymphoid cells into bioactivated dermal skin substitute materials.

### Analysis of the revascularization capacity using CD31

**Figure 4.**
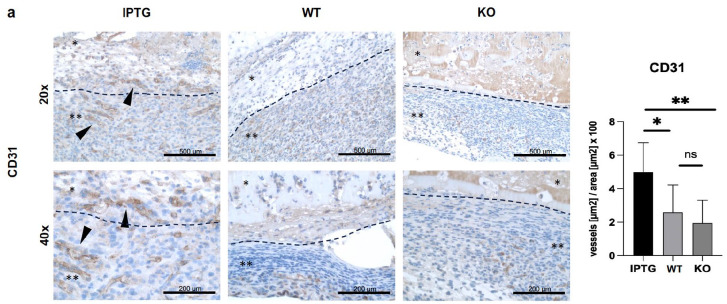
Immunohistological staining of cell migration into scaffolds colonized with either IPTG-simulated transgenic cyanobacteria or wild-type bacteria (WT). The control scaffolds (KO) contained no bacteria. The scale bar represents 500 μm at 20× magnification and 200 μm at 40× magnification. Representative images are shown. The mark with one star indicates the scaffold. Two stars mark the adherent ingrowing tissue layer. (a) Quantification of cell density (cells per square millimeter) in the scaffolds stained for LYVE-1, analyzed using Image-J. (b) Quantification of cell density (cells per square millimeter) in the scaffolds stained for VEGFR-3, analyzed using Image-J. The black dashed line marked the scaffolds boundary and transition into the surrounding tissue. Statistical analysis (*N* ⩾ 9, ns *p* > 0.05; *p ⩽ 0.05; **p ⩽ 0.01; ***p ⩽ 0.001).

Next, we investigated the impact of bioactivated scaffolds on endothelial cells. Employing the mouse wound model as previously described, the scaffolds were utilized and subsequently subjected to immunohistological examination targeting the CD31 marker. CD31, a specific marker for endothelial cells, facilitated the detection of vessel density within the scaffold, enabling conclusions to be drawn regarding revascularization.^33^ Therefore, the endothelial cells were immunostained, and the vascular area was subsequently recorded in relation to the total area in μm^2^ at 20× magnification. Immunohistological staining unveiled a significantly heightened vessel area within the scaffolds harboring transgenic cyanobacteria (512 ± 178 μm^2^) in comparison to those colonized with wild-type bacteria (224 ± 162 μm^2^) and the control (189 ± 158 μm^2^). Notably, the presence of microbial cells within the scaffolds containing wild-type bacteria exhibited no significant difference compared to the control scaffold devoid of bacteria, suggesting a key influence of secreted hyaluronic acid on endothelial cell migration (Figure 4).

### Scaffold migration of inflammatory cells

**Figure 5.**
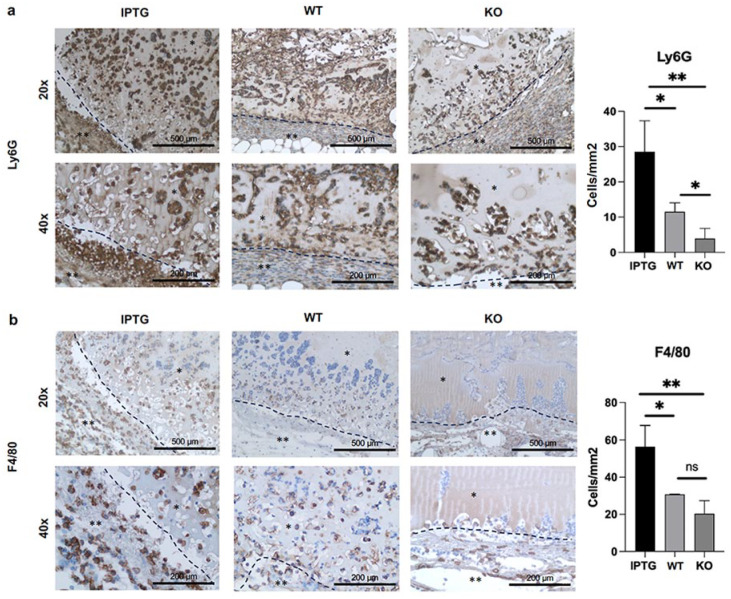
Immunohistological assessment of inflammatory cell infiltration into scaffolds colonized with either IPTG-induced transgenic cyanobacteria or wild-type bacteria (WT). Control scaffolds (KO) were devoid of bacterial colonization. The scale bar represents 500 μm at 20× magnification and 200 μm at 40× magnification. Representative images are depicted. The mark with one star indicates the scaffold. Two stars mark the adherent ingrowing tissue layer. (a) Quantification of neutrophil and monocyte cell density in cells per square millimeter within scaffolds stained with the Ly6G marker, analyzed using ImageJ. (b) Quantification of macrophage cell density in cells per square millimeter within scaffolds stained with the F4/80 marker, analyzed using ImageJ. The black dashed line marked the scaffolds boundary and transition into the surrounding tissue. Statistical analysis: (*N* ⩾ 9, ns *p* > 0.05; *p ⩽ 0.05; **p ⩽ 0.01).

During the wound healing process, numerous immune cells are recruited. It is therefore particularly important to analyze the immune cells migrating into the scaffold. Here we differentiate between cells of the early inflammatory phase, which includes monocytes and granulocytes (neutrophils and eosinophils), and late inflammation after 48–72 h, characterized by the immigration of macrophages for phagocytosis. For this investigation, two crucial markers of inflammatory cells were examined. Ly6G, a 25-kDa GPI protein, serves as a reliable marker for peripheral neutrophils, monocytes, and granulocytes detection.^34,35^ The F4/80 antigen, on the other hand, is expressed in various mature tissue macrophages.^34,36^ Immunohistological analysis revealed an elevated migration of Ly6G-stained cells into bioactivated scaffolds (Figure 5(a)). Using ImageJ, we quantified a significant increase, measured as cells per mm^2^, of Ly6G-stained neutrophils and monocytes in scaffolds colonized with transgenic cyanobacteria (28 ± 9 cells/mm^2^) at 20× magnification compared to those with wild-type bacteria (11 ± 3 cells/mm^2^) or the control (4 ± 3 cells/mm^2^). Furthermore, a notable increase in inflammatory cells was observed in skin substitute materials hosting wild-type bacteria compared to the control. This observation suggests that bacteria recognized for their compatibility with humans exert a stimulatory effect on the local inflammatory process.

A parallel trend was observed in the immunohistochemical staining of F4/80 (Figure 5(b)). Similarly, an augmented migration of macrophages, quantified as cells per mm^2^, was noted in scaffolds containing genetically modified cyanobacteria (56 ± 12 cells/mm^2^) at 20× magnification in contrast to those with wild-type bacteria (30 ± 2 cells/mm^2^) and the control (19 ± 9 cells/mm^2^). Nonetheless, no significant disparities were observed between scaffolds colonized with wild-type bacteria and the control.

## Discussion

The study investigated the effects of bioactivated scaffolds containing cyanobacteria on tissue regeneration and vascularization. Scaffolds colonized with bacteria showed significantly enhanced tissue formation and vascularization compared to controls, attributed to the oxygen and hyaluronic acid produced by the bacteria. Histological analysis indicated increased cell migration, particularly of lymphatic endothelial cells. We also observed an immune response, especially in scaffolds with transgenic bacteria. Overall, the study highlights the potential of cyanobacteria in improving wound healing and angiogenesis through their bioactive properties.

Hypoxia and the absence of growth factors persist as unresolved challenges in tissue engineering, particularly in the management of chronic wounds.^7,37^ Numerous strategies have been devised to address this deficiency. Various oxygen carriers, such as perfluorocarbon or modified hemoglobin, have been utilized, albeit with the drawback of a finite oxygen supply.^38^ To address the need for adequate oxygen and growth factor delivery to non-vascularized scaffolds, innovative models like the arteriovenous (AV) loop model have emerged.^39,40^ However, implementation of this model necessitates intricate surgical procedures, including vascularization, rendering its application in tissue engineering challenging.

A prospective solution aimed at mitigating hypoxic tissue conditions by employing photosynthetic microorganisms as a sustained source of oxygen was first pioneered by Hopfner et al.^41^ and Schenck et al.^32^ Their innovative approach involved incorporating the algal strain *Chlamydomonas reinhardtii* into collagen-based 3D scaffolds, which were meticulously scrutinized both in vitro and in vivo. Notably, upon light exposure, a marked augmentation in vascularization levels was observed post-implantation of the colonized scaffolds in a murine model. Extending this line of inquiry, the viability of employing the photosynthetic euryhaline unicellular cyanobacterium *Synechococcus sp. PCC 7002* (Syn 7002) was explored. Its rapid growth rate, resilience to elevated light intensities, high salinity, and wide temperature ranges substantiated this choice.^42,43^ Furthermore, the complete sequencing of its genome, coupled with ongoing advancements in molecular manipulation techniques, renders it an auspicious candidate for diverse biotechnological applications.^44^

Previous investigations attested to the biocompatibility of these cyanobacteria with human dermal cells, thus underscoring their suitability for prospective tissue engineering endeavors.^45^ Furthermore, the “generally regarded as safe” (GRAS) classification bestowed upon most of these photosynthetic microorganisms underscores their suitability for applications spanning dermal dressings, skincare formulations, and food additives, owing to their absence of human pathogens such as viruses, prions, or bacterial endotoxins.^46,47^

Given their inherent properties, cyanobacteria present a new approach for producing recombinant proteins, including cytokines and growth factors. Photosynthetic cyanobacteria are experiencing a surge in recognition within the realm of regenerative medicine. Their regenerative capabilities are expanding beyond bone tissue regeneration^48^ to encompass the restoration of muscle functionality and regeneration.^49^ Inspired by the notion that genetic modifications could potentiate regenerative capacities, Chávez et al.^31^ introduced the concept of leveraging transgenic cyanobacteria as a biotechnological platform for secreting recombinant human growth factors alongside oxygen to stimulate lymphangiogenesis in vitro. Genetic manipulation facilitated the overexpression of hyaluronic acid synthase in cyanobacteria, leading to the secretion of hyaluronic acid into the scaffolds, thereby fostering increased gene and protein expression of essential lymphatic markers and inducing lymph vessel formation in vitro.

Having demonstrated the stimulatory potential of these scaffolds in vitro, this study now turns to evaluating their regenerative efficacy in vivo, particularly in a murine wound model. Employing commercial collagen scaffolds from Integra, an FDA-approved medical device company, the study investigates the colonization of these scaffolds with cyanobacteria to elucidate their role in augmenting wound healing. Bilaterally implanted in a full-thickness wound model in mice, the colonized scaffolds were retrieved after 7 days for histological examination of the ingrowing tissue. The central hypothesis posits that through photosynthetic oxygen production and hyaluronic acid secretion, transgenic cyanobacteria significantly contribute to tissue regeneration.

First, the adjacent tissue layer was meticulously assessed to ascertain the stimulatory impact on the surrounding tissue. To enhance comparability and reduce reliance on examiner preparation skills, a dye-free, non-absorbable monoprene mesh containing polypropylene (PP) filaments was introduced into the wound prior to scaffold implantation. A precise dissection was performed beneath the mesh for explantation purposes. Upon explantation of the scaffolds, an adherent vascularized tissue layer was observed between the scaffolds colonized with bacteria and the inserted Monoprolen Mesh (see Figure 1). This phenomenon was absent in the control scaffolds, suggesting that the oxygen released by the bacteria stimulates tissue regeneration. Transillumination facilitated the evaluation of tissue thickness and vascularization, revealing a robustly vascularized layer enveloping the colonized scaffolds after 7 days, contrasting with scant tissue formation in the control group. Additionally, a quantification of vascular area using the Veg-Seg tool further accentuated the enhanced vascularization observed in scaffolds colonized with transgenic bacteria compared to those with wild-type bacteria, thereby bolstering the study’s findings. Sufficient oxygen production by the cyanobacteria was previously validated in the preliminary in vitro investigations.^31,45^ Depending on the bacterial concentration, oxygen levels exceeding 50% pO_2_ were attained under constant illumination during a measurement period exceeding 30 h.^11,14^ In the context of the in vivo experiments, illumination followed a circadian rhythm (day/night), thereby suggesting a diminished oxygen production owing to intermittent illumination. The changing light conditions create an inconstant oxygen production, so that an increased formation of harmful ROS is unlikely. Nevertheless, the bacteria serve as an oxygen reservoir independent of blood perfusion, potentially mitigating hypoxic conditions, particularly in tissue engineering contexts characterized by low perfusion or in chronic wounds. In addition, the oxygen produced counteracts anaerobic bacterial infections and could, therefore, reduce the risk of infection, particularly in the case of contaminated wounds.

The second stage encompassed histological processing of the scaffolds. Initially, we scrutinized cell migration into the scaffolds, emphasizing the depth of infiltration in µm regardless of cell phenotype. Scaffolds loaded with cyanobacteria exhibited a conspicuous escalation in migration, potentially attributed to the oxygenic advantage conferred by these scaffolds. Moreover, discernible disparities emerged between scaffolds colonized by transgenic and wild-type bacteria, intimating an additional impact of the secreted hyaluronic acid. Throughout a 7-day observation period, only marginal degradation of the fibrin scaffold was discerned, possibly hindering cellular migration. Encapsulation of cyanobacteria within fibrin was executed utilizing the seeding technique pioneered by Schenck et al.,^32^ aiming to confine bacteria within the scaffolds and preclude contamination. Fibrin fixation not only facilitated unimpeded oxygen release and medium exchange in our preliminary assessments but also attenuated the host immune response when employing other microorganisms.^50^ It should be noted that fibrin glue itself can lead to a reduction in gene and protein expression even when used in tissue engineering approaches in previous experiments.^51^

Next, we analyzed the phenotype of the migrated cells, with an initial emphasis on lymphatic vessel regeneration. The hyaluronic acid secretion by bacteria serves as a pivotal growth factor for lymphatic vessel formation, inducing lymphangiogenesis through binding to the LYVE-1 receptor.^12,52,53^ In our in vitro experiments, we successfully demonstrated a stimulating effect on gene and protein expression, as well as lymphatic vessel formation in the tube formation assay, mediated by bacterial-secreted hyaluronic acid.^31^ It is now of particular interest to examine the impact of bacterial-derived hyaluronic acid on lymphangiogenesis stimulation in the animal model. Immunohistological staining of LYVE-1 and VEGFR-3 markers specific to lymphatic endothelium was conducted to identify lymphatic endothelial cells (LECs).^54,55^ Scaffolds hosting transgenic bacteria stimulated with IPTG exhibited a significantly higher migration rate concerning LYVE-1 and VEGFR-3 compared to scaffolds with wild-type bacteria. The augmented detection of lymphatic markers implies heightened cell migration induced by bacterial hyaluronic acid production. As a crucial component of the extracellular matrix, hyaluronic acid plays a pivotal role in regulating cell functions and cell-cell communication, as evidenced in other studies. Hakki et al.^56,63^ demonstrated in cementoblasts that hyaluronic acid enhances cell migration, viability, and mineralized tissue-specific gene expression, whereas McLaughlin exhibited decreased endometrial cell attachment, migration, and invasion through hyaluronic acid synthesis inhibition.^64^ Accordingly, bacterially secreted hyaluronic acid in the wound region can significantly influence cell migration, particularly of lymphatic cells, potentially serving as a key factor in regenerating chronic wounds characterized by a compromised lymphatic vessel network.^57–59^

Furthermore, we conducted histological examinations to assess the revascularization capacity of the bioactivated scaffolds in a mouse model. Endothelial cells were identified using the transgenic marker CD31, and the area of formed vessels in square micrometers (µm^2^) was analyzed relative to the total scaffold area. Scaffolds bioactivated with transgenic bacteria exhibited a significantly higher vessel count compared to scaffolds hosting wild-type bacteria and control scaffolds. This finding suggests that the secreted hyaluronic acid exerts an activating influence on angiogenesis and blood vessel revascularization. A similar effect was observed by Liu et al.,^60^ who employed a hyaluronan-collagen hydrogel to enhance angiogenesis through exosome integration for traumatic brain injury repair. Interestingly, the literature indicates that the stimulatory effect of hyaluronic acid varies depending on its molecular weight.^12^ Low molecular weight hyaluronic acid exerts a positive influence on angiogenesis and lymphangiogenesis, and has been shown to stimulate vascular endothelial cell proliferation, migration, and sprouting formation both in vitro and in various in vivo angiogenesis models.^61^ In contrast, hyaluronic acid with a molecular weight exceeding 1 MDa appears to exhibit anti-angiogenic and anti-lymphangiogenic effects.^61,62^ The molecular size of the secreted HA measured by chromatography revealed a peak at >1 MDa for strains HA08 and HA12, with HA13 and HA01 showing the largest peak between 2 and 2.2 MDa.^22^ The results indicate a positive effect on angiogenesis and lymphangiogenesis, suggesting the presence of sufficiently short-chain HA molecules to support this process. Various studies describe the involvement of hyaluronidases in inflammatory processes. Thus, HA has a short half-life during inflammation and undergoes rapid turnover into short low-molecular HA fragments.^63–65^ Accordingly, cyanobacteria bioactive scaffolds have great potential to stimulate angiogenesis and the migration of endothelial cells through the secreted hyaluronic acid and the oxygen produced.

The implantation of a foreign object and the introduction of bacteria into a healthy mammalian host elicit an inflammatory response. To assess the extent of the local inflammatory process, we conducted histological analyses focusing on key representatives of local inflammatory reactions. During wound healing, numerous immune cells are recruited, with a distinction drawn between an early inflammatory phase and a late inflammatory phase.^66^ The early phase involves monocytes and granulocytes (neutrophils and eosinophils), while the late phase, occurring after 48–72 h, is characterized by the infiltration of macrophages for phagocytosis. Ly-6G, a 25-kDa GPI protein, serves as a reliable marker for detecting peripheral neutrophils, monocytes, and granulocytes.^67^ In contrast, the F4/80 antigen is expressed in various mature tissue macrophages.^36^ The relatively low number of inflammatory cells observed in the control group is likely attributable to a foreign body reaction. Similar observations were made by Cao et al.^68^ who formulated three-dimensional scaffolds composed of poly-lactic-co-glycolic acid (PLGA) interconnected via an arteriovenous (AV) loop and subsequently implanted beneath the inguinal skin of rats. Histological examination revealed a predominant foreign body giant cell response and diminished vascularization during the period of tissue ingrowth into the scaffolds, spanning from 2 to 8 weeks post-implantation. Conversely, a significantly elevated number of cells expressing the Ly-6G and F4/80 markers, measured in cells per square millimeter (cells/mm^2^), was noted in scaffolds colonized by bacteria compared to those devoid of bacteria. This reaction suggests that despite the fibrin coating, the mouse responds to the bacteria with local inflammation. Substantially increased values were detected in scaffolds hosting transgenic bacteria, which may be attributed to the secretion of hyaluronic acid. Cyanobacteria predominantly produce and secrete short-chain polysaccharide length hyaluronic acid. Low molecular weight hyaluronic acid has the ability to bind to Toll-like receptors (TLRs), initiating a signaling cascade that induces the production of proinflammatory cytokines and chemokines in various cell types.^69^ Studies by Iwata and others have demonstrated that low molecular weight hyaluronic acid stimulates B lymphocytes to produce IL-6 and TGF-beta via the TLR4 receptor.^70^ Additionally, Bourguignon et al.^71^ have shown that small hyaluronic acid fragments promote the association of CD44 with TLR2, TLR4, and MyD88, resulting in NF-κB-specific transcriptional activation and the expression of proinflammatory cytokines IL-1β and IL-8 in human breast cell lines. In summary, the local inflammatory response can be primarily attributed to a foreign body reaction and the secretion of low molecular weight hyaluronic acid, acting as a catalyst for local inflammation. Nonetheless, additional investigations are warranted to assess potential systemic effects in the mouse model.

Even though we were able to demonstrate the successful application of bioactivated scaffolds using a mouse model, there are limitations to this study. Several limitations warrant consideration. First, considering animal welfare, we deliberately selected a study time point of 7 days post-implantation, which consequently imposes a limitation on the study. Additionally, the animals utilized were healthy mice without any impairments in wound healing, thereby restricting the applicability of our findings to chronic wound healing scenarios. Therefore, it is of significant interest to extend the study duration in future research and to adapt the mouse wound model to reflect chronic wound conditions better. Next, cyanobacteria’s dependence on light limits their therapeutic potential to easily accessible or surface wounds, as achieving sufficient light exposure in deeper tissues remains challenging. The potential for prolonged immune tolerance in diverse patient populations, especially those with immunocompromised conditions, requires further investigation. While preliminary animal studies are promising, more extensive safety and efficacy trials are essential to evaluate the long-term impact and optimize the dosage, duration, and delivery methods of cyanobacterial biomaterials in clinical settings. Nevertheless, photosynthetic biomaterials, potentially combined with genetic engineering, could enhance tissue regeneration and provide stable oxygenation to non-perfused organs. However, the transition from basic scientific research into clinical practice will require input from experts in public policy, ethics, and economics.

## Conclusion

This study demonstrates that photosynthetic biomaterials, specifically bioartificial scaffolds with genetically modified cyanobacteria (*Synechococcus* sp. PCC 7002), have the potential to enhance chronic wound healing. The scaffolds promote local tissue oxygenation and secrete lymphangiogenic hyaluronic acid, crucial for a healing environment. Successful scaffold colonization, increased vascularization, and cell migration were observed, along with upregulation of lymphatic (LYVE-1, VEGFR3) and endothelial markers (CD31), and modulation of inflammatory responses (Ly6G, F4/80). This innovative approach shows promise for improving chronic wound treatment and patient outcomes, warranting further optimization and investigation for clinical application.
